# Headache and migraine clinical practice guidelines: a systematic review and assessment of complementary and alternative medicine recommendations

**DOI:** 10.1186/s12906-021-03401-3

**Published:** 2021-09-22

**Authors:** Jeremy Y. Ng, Christina Hanna

**Affiliations:** Department of Health Research Methods, Evidence, and Impact, Faculty of Health Sciences, McMaster University, Michael G. DeGroote Centre for Learning and Discovery, Room 2112, 1280 Main Street West, Hamilton, Ontario L8S 4K1 Canada

**Keywords:** Headache, Migraine, Complementary and alternative medicine, Systematic review, AGREE II, Clinical practice guideline

## Abstract

**Background:**

Globally, 3 billion people suffer from either migraine or tension-type headache disorder over their lifetime. Approximately 50% of American adults suffering from headache or migraine have used complementary and alternative medicine (CAM), however, the quality and quantity of recommendations associated with such therapies across clinical practice guidelines (CPGs) for the treatment and/or management of these conditions are unknown. The purpose of this study was to identify the quantity and assess the quality of such CAM recommendations.

**Methods:**

MEDLINE, EMBASE and CINAHL were systematically searched from 2009 to April 2020; the Guidelines International Network and the National Center for Complementary and Integrative Health websites were also searched for eligible CPGs. CPGs were included if they provided any therapy recommendations. Eligible CPGs included those written for adult patients with headache and migraine; CPGs containing CAM recommendations were assessed twice for quality using the AGREE II instrument, once for the overall CPG and once for the CAM sections.

**Results:**

Of 486 unique search results, 21 CPGs were eligible and quality assessed; fifteen CPGs mentioned CAM, of which 13 CPGs made CAM recommendations. The overall CPG assessment yielded higher scaled domain percentages than the CAM section across all domains. The results from highest to lowest were as follows (overall, CAM): clarity of presentation (66.7% vs. 50.0%), scope and purpose (63.9% vs. 61.1%), stakeholder involvement (22.2% vs. 13.9%), rigour of development (13.5% vs. 9.4%), applicability (6.3% vs. 0.0%), and editorial independence (0.0% vs. 0.0%).

**Conclusions:**

Of the eligible CPGs, the CAM sections were of lower quality compared to the overall recommendations across all domains of the AGREE II instrument. CPGs that scored well could serve as a framework for discussion between patients and healthcare professionals regarding use of CAM therapies in the context of headache and migraine.

**Supplementary Information:**

The online version contains supplementary material available at 10.1186/s12906-021-03401-3.

## Background

The prevalence of headache disorders is increasing globally [1]. In 2016, it was estimated that 3 billion people worldwide suffered from either migraine or tension-type headache disorder, with disability adjusted life years approximately 1.9 and 0.3% respectively [1, 2]. Headache, one of the most prevalent conditions in the world, can be associated with more severe primary headache disorders, such as migraine and tension-type disorders [3]. Clinicians regularly consult the International Classification of Headache Disorders (ICHD) to classify and diagnose specific headache disorders, such as migraine, tension-type, and cluster headache [4]. The different types of headache disorders are defined, diagnosed and screened for according to the ICHD, currently in its third edition published in 2018 [4], following the publication of the first two editions [5, 6]. This first version was published in 1988 and mainly based on expert opinions, while the ICHD-II published in 2004 contained a variety of improvements, partly due to new research and partly due to updated expert opinions. Prior to this, the Headache Classification Committee of the International Headache Society released an ICHD-3 beta version in 2013 ahead of the ICHD-3 [7]; at that time, the main reason for this was to synchronize the ICHD-3 with the World Health Organization’s (11th edition) of the International Classification of Diseases (ICD-11) [8], however, based on delays at the WHO, the former was published ahead of the latter [4]. New scientific evidence played a comparatively greater role in the improvements made to the ICHD-3 beta, and all other changes included in the ICHD-3 were based on this evidence. Since this time, it has been found that the ICHD-3 is significantly more specific than the ICHD-3 beta for the diagnosis of migraine with aura and with typical aura [4, 8, 9].

The prevalence of headaches globally has resulted in significant costs and impacts on society as a whole. A review of studies evaluating the quality of life in patients with primary headache disorders indicated that the health-related quality of life for patients suffering from primary headache disorders, such as migraine and cluster headache, was consistently lower than that of the general population [10]. In addition, an American study identified that the average healthcare expenditure of Americans suffering from migraine was significantly higher than those of non-migraine sufferers [11]. Standard treatments for headache disorders include nonsteroidal anti-inflammatory drugs (NSAIDs) and acetaminophen, but there is also an increasing interest among patients in using complementary and alternative medicine (CAM) [12]. According to the National Center for Complementary and Integrative Health (NCCIH), "complementary medicine" is defined as non-mainstream healthcare approaches that are used together with conventional medicine, while "alternative medicine" is defined as non-mainstream healthcare approaches that are used in place of conventional medicine [13, 14].

The main reason that patients use CAM therapies is to avoid the side effects associated with conventional medicine [15]. It was found that the approximate prevalence of CAM use among American adults suffering from migraine was 50%, which is a significantly higher proportion than non-migraine suffering adults who seek CAM therapies [16, 17]. Some common CAM therapies used for headache disorders include acupuncture, massage, chiropractic, and herbal and dietary supplements [15]. To date, some research has found the effectiveness of certain CAM therapies to be promising. In two large clinical trials, it was found that a greater number of individuals exposed to acupuncture experienced at least a 50% reduction in headaches when compared to the control group [18, 19]. Another study discovered that participating in massage therapy, which targets muscular trigger points for chronic tension headaches, reduced the frequency of chronic tension headaches per week when compared to the baseline frequency [20].

Despite the fact that some CAMs are supported by promising evidence, many clinicians lack training on these therapies for the treatment/management of headache and migraine, which may result in them recommending them less frequently [21]. One survey found that only about 25% of American medical students, residents, and clinicians received training in CAM as part of their education [21]. In addition, many physicians do not mention CAM resources in their discussions with patients, and many patients do not report their CAM use [21]. Healthcare professionals routinely consult evidence-based clinical practice guidelines (CPGs) to identify therapy recommendations and their associated risks and benefits. Using the information from CPGs, healthcare professionals can address patient concerns and needs to inform discussions surrounding shared decision-making. Most of the treatments administered for headache and migraine according to CPGs, such as the U.S. Headache Consortium guidelines, include recommendations about NSAIDs and triptans, among other pharmacological therapies [22]. However, CAM recommendations may be included less frequently or inconsistently across CPGs, based on the fact that there is generally a lower quantity and quality of randomized controlled trials and observational studies forming the evidence-base for these types of therapies [23, 24]. The purpose of this study is to conduct a systematic review to determine the mention of CAM therapies in CPGs for the treatment and/or management of headache and migraine, and assess the quality of CAM recommendations using the AGREE II instrument.

## Methods

### Approach

To identify CPGs for the treatment and/or management of headache and migraine, a systematic review was conducted using standard methods [25] and Preferred Reporting Items for Systematic Reviews and Meta-Analyses (PRISMA) criteria [26]. A protocol was registered with PROSPERO, registration number CRD42020182233. The widely-used and validated Appraisal of Guidelines for Research and Evaluation II (AGREE II) instrument [27] was used to assess the CPGs containing CAM recommendations; twenty-three items comprise the instrument. These items are grouped into six domains, each designed to assess different aspects of CPGs quality, as follows: scope and purpose, stakeholder involvement, rigor of development, clarity and presentation, applicability, and editorial independence. CPGs containing CAM recommendations were assessed twice with the AGREE II instrument: once for the overall CPG, and once for only the CAM section of the CPG.

### Eligibility criteria

The Population, Intervention, Comparison and Outcomes (PICO) framework was used to identify the eligibility criteria for headache and/or migraine CPGs. The population included adults aged 19 years and older with headache and/or migraine. The interventions included evidence-based CPGs that provided treatment and/or management recommendations for headache and/or migraine. From these eligible CPGs, we determined whether they included any mention or recommendations of CAM therapies. Comparisons referred to the assessed overall quality of headache and/or migraine CPGs and the CAM recommendation sections using the AGREE II instrument. The AGREE II scores were the outcomes, reflecting CPG content and format. Additionally, CPGs were restricted to those as follows: developed by non-profit organizations including disease-specific foundations, government agencies, academic institutions or professional associations or societies; published in 2009 to 2020; published in English; and either available publicly or by order through our library system. Ineligible publications included: consensus statements, protocols, abstracts, conference proceedings, letters or editorials; based on primary studies that evaluated headache management or treatment; or focused on headache education, curriculum, research, training, professional certification or performance. The methods used to guide the development of the CPGs were considered when searching and screening for CPGs meeting our eligibility criteria. We specifically included only evidence-based CPGs as they provided recommendations based on a systematic literature search for evidence, as opposed to solely expert opinion or consensus-based CPGs which are reflective of a lower quality of evidence. The only exception for inclusion included the case whereby a CPG was informed by evidence, however, expert opinion was used to formulate a recommendation for certain therapies for which the available evidence-base was lacking.

### Searching and screening

The search was conducted on April 17, 2020, from 2009 to April 16, 2020 inclusive, on MEDLINE, EMBASE and CINAHL. The search strategy (Supplementary File 1) included indexed headings and keywords that reflected terms commonly used in the literature to refer to headache and migraine. The Guidelines International Network, a repository of guidelines [https://www.g-i-n.net/], was also searched using keywords, including “headache” and “migraine”. Next, the NCCIH website which contained a single list of CAM guidelines was searched [https://nccih.nih.gov/health/providers/clinicalpractice.htm]. CH and another research assistant screened titles and abstracts from all sources, and they confirmed eligibility by screening full-text items. JYN reviewed the screened titles and abstracts and full-text items to standardize screening, and helped to resolve selection differences between the two screeners (CH and the other research assistant) through discussion.

### Data extraction and analysis

CH and the other research assistant data extracted the following items from each eligible CPG: date of publication; country of first author; type of organization that published the CPG (academic institutions, government agencies, disease-specific foundations, or professional associations or societies); and whether any CAMs were mentioned in this CPG. After determining if CAMs were mentioned in a CPG, the types of CAM mentioned, CAM recommendations made, CAM funding sources, and whether any CAM providers were part of the CPG panel were also data extracted. For the purpose of this review, we defined a CAM funding source as that which was provided by a CAM research organization or CAM professional association. Each CPG developer’s website was also searched to identify any associated knowledge-based resources in support of implementation. For eligible CPGs that did not contain CAM therapy recommendations, only demographic information was collected.

### Guideline quality assessment

Standardized methods for applying the AGREE II instrument were followed for the extraction and analysis of data from eligible CPGs containing CAM recommendations [27]. The first step involved conducting a pilot test of the AGREE II instrument; JYN, CH and the other research assistant independently assessed three separate CPGs with the AGREE II instrument, then they met to discuss and resolve any discrepancies. Next, CH and the other research assistant independently assessed all eligible CPGs containing CAM therapy recommendations twice (i.e. once for the overall CPG, and once for the CAM sections of the CPG). CPGs were assessed according to the 23 AGREE II items comprised of 6 domains using a seven-point Likert scale from strongly disagree (1) to strongly agree (7). Using the information from these scores, we recommended for or against the use of each CPG. Supplementary File 2 includes the modified AGREE II items that were used to guide the scoring of the CAM sections of each CPG. Any discrepancies in scores between the two assessors were resolved by JYN. To calculate average appraisal scores, we took the average rating for all 23 items of a single appraiser of a single CPG, followed by taking the average of this value for both appraisers. The average of both appraisers’ “overall guideline assessment” scores for each CPG were calculated to provide the average overall assessment score. Calculating the scaled domain percentages required the addition of both appraisers' ratings of items within each domain, and scaling by maximum and minimum possible domain scores before converting this value into a percentage. The scaled domain percentages were generated for inter-domain comparison. Tabulation of the average appraisal scores, average overall assessments, and scaled domain percentages for each CPG was used for comparison.

## Results

### Search results (Fig. 1)

Searches retrieved 536 items, 486 of which were unique. After screening for eligibility, 461 titles and abstracts were eliminated. Of the 23 full-text articles, two were not eligible, because the CPG was irretrievable (*n* = 1), or the CPG was a summary (*n =* 1), leaving 21 CPGs eligible for review [28–48]; thirteen CPGs made CAM recommendations [28, 29, 32, 34–36, 38–41, 43–45, 48], two CPGs made mention of CAM but provided no recommendations [33, 48], and the final six CPGs made no mention nor recommendations pertaining to CAM [30, 31, 37, 42, 46, 47]. The citations associated with the excluded full-text items are provided in Supplementary File 3.

**Fig. 1:**
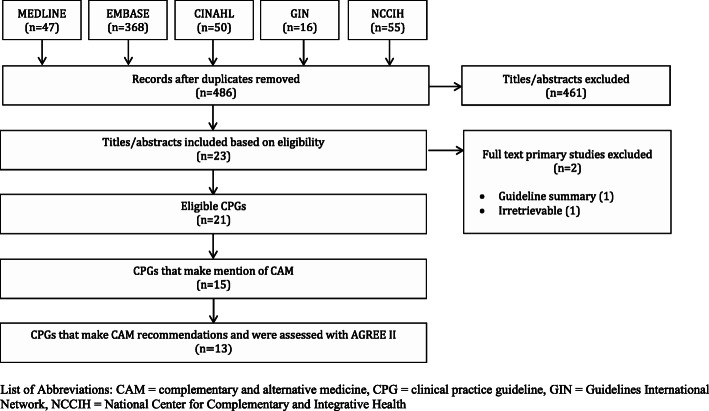
PRISMA Diagram

### Guideline characteristics (Table 1)

Eligible CPGs were published from 2009 to 2020 in the USA (*n* = 5), Canada (*n* = 4), Germany (*n* = 2), Italy (*n* = 2), China (*n* = 1), Croatia (*n* = 1), Denmark (*n* = 1), France (*n* = 1), Japan (*n* = 1), Qatar (*n* = 1), Scotland (only) (*n* = 1) and the UK (*n* = 1). The CPGs were funded and/or developed by academic (*n* = 2) and professional (*n* = 19) associations or societies. Fifteen CPGs made mention of CAMs, with all 15 CPGs mentioning CAM therapies for different headache disorders, including tension-type, migraine and cluster headaches [28, 29, 32–36, 38–41, 43–45, 48]. These CAMs included dietary supplements (e.g. magnesium, coenzyme Q10, melatonin) (*n* = 12), herbal medicine (e.g. butterbur, feverfew) (*n* = 9), oxygen therapy (including hyperbaric, 100%/pure) (*n* = 9), electrotherapy (e.g. transcutaneous electrical and nerve stimulation (TENS) (*n* = 7), acupuncture (*n* = 6), behavioural therapy (e.g. relaxation, cognitive behavioural therapy, hypnosis) (*n* = 6), manual therapy (e.g. spinal manipulation, massage) (*n* = 3), homeopathy (*n* = 2) and Chinese medicine (*n* = 1). Of the 15 CPGs, recommendations relating to CAM were made in 13 CPGs; only these CPGs were assessed using the AGREE II instrument. CAM funding sources were used in 5 of the CPGs [28, 29, 32, 36, 45], and 3 CPGs included CAM providers as part of the CPG panel [28, 36, 45].

**Table 1:** Characteristics of eligible guidelines

Guideline	Country (First Author)	Developer	CAM Category	Guideline Topic
Ren 2020 [28]	China	China Association of Chinese Medicine	Acupuncture, herbal therapy, Chinese medicine	Diagnosis and treatment for headache
Araki 2019 [29]	Japan	Japanese Society of Neurology and Japanese Headache Society, with collaboration from the Japanese Society of Neurological Therapeutics and the Japan Neurosurgical Society	Acupuncture, herbal, electrotherapy, dietary supplements, oxygen therapy, behavioural therapy	Chronic headache
Sacco 2019 [30]	Italy	European Headache Federation	None	Migraine prevention
Scottish Intercollegiate Guidelines Network 2018 [31]	Scotland	Scottish Intercollegiate Guidelines Network	None	Pharmacological management of migraine
Al Khaled 2017 [32]	Qatar	Ministry of Public Health of Qatar	Acupuncture, herbal therapy, homeopathy, electrotherapy, dietary supplements, oxygen therapy, behavioural therapy	Headache in adults
Orr 2016 [33]	USA	American Headache Society	Dietary Supplements	Management of adults with acute migraine in the emergency department
Moisset 2016 [34]	France	French society for the Study of Migraine and Headache Disorders (SFEMC1) and the French Society of Neurology (SFN2)	Oxygen therapy	Emergency management of headache
Robbins 2016 [35]	United States	American Headache Society	Electrotherapy, dietary supplements	Treatment of cluster headache
Becker 2015 [36]	Canada	Canadian Family Physician, Alberta College of Family Physicians	Herbal therapy, electrotherapy, dietary supplements, oxygen therapy, behavioural therapy	Primary care management of headache in adults
Worthington 2013 [37]	Canada	The Canadian Journal of Neurological Sciences	None	Acute drug therapy for migraine headache
Bendtsen 2012 [38]	Denmark	Danish Headache Society	Acupuncture, dietary supplements	Diagnosis and treatment of headache disorders and facial pain
Holland 2012 [39]	United States	American Academy of Neurology	Herbal therapy, dietary supplements, oxygen therapy	NSAIDs and complementary treatments for episodic migraine prevention
NICE 2012 [40]	United Kingdom	National Institute for Health and Care Excellence	Dietary supplements, oxygen therapy	Diagnosis and management of headache
Pringsheim 2012 [41]	Canada	The Canadian Neurological Society	Herbal therapy, dietary supplements	Migraine prophylaxis
Silberstein 2012 [42]	USA	American Academy of Neurology	None	Pharmacologic treatment for episodic migraine prevention in adults
Sarchielli 2012 [43]	Italy	Italian Society for the Study of Headaches	Acupuncture, manual therapy, herbal therapy, electrotherapy, dietary supplements, oxygen therapy behavioural therapy	Guidelines for primary headaches
Vukovic 2012 [44]	Croatia	Croatian Society for Neurovascular Disorders, Croatian Medical Association	Acupuncture, manual therapy, herbal therapy, homeopathy, electrotherapy, dietary supplements, oxygen therapy, behavioural therapy	Treatment of primary headache
Bryans 2011 [45]	Canada	Canadian Chiropractic Protective Association	Manual therapy (i.e. chiropractic), electrotherapy, behavioural therapy	Chiropractic treatment of adults with headache
Evers 2011 [46]	Germany	EFNS Guidelines	None	Treatment of medication overuse headache
Saper 2010 [47]	USA	Michigan Head-Pain & Neurological Institute	None	Continuous opioid therapy for refractory daily headache
Evers 2009 [48]	Germany	European Federation of Neurological Societies (EFNS)	Herbal therapy, dietary supplements	Drug treatment of migraine

### Guidelines mentioning CAM without recommendations

Of 21 eligible CPGs, two CPGs made mention of CAM without making recommendations [33, 48]. The CAMs mentioned included magnesium, butterbur root extract, feverfew (*Ternacetum parthenium*), riboflavin, and coenzyme Q10. In one CPG, there was a detailed description of various experimental studies about the impact of magnesium on relieving headache, but there was a clear statement that the authors were not making a recommendation for magnesium use [33]. The other CPG had described a variety of herbal treatments, but vaguely [48].

### CAM therapies with recommendations across assessed CPGs

**Fig. 2:**
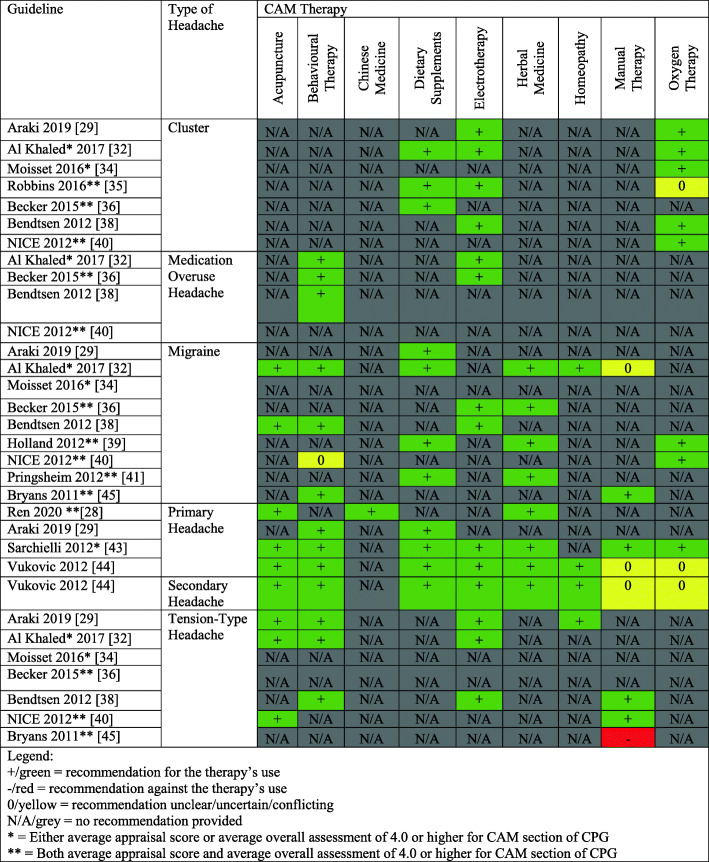
Summary of CAM Recommendations in Clinical Practice Guidelines

We provide a summary of CAM recommendations made across headache and migraine CPGs for the benefit of clinicians and researchers in Fig. 2. Of the 13 included CPGs, the most commonly recommended CAM therapies were dietary supplements, which were recommended by 10 CPGs, followed by oxygen therapy, herbal medicine, electrotherapy, acupuncture, behavioural therapy, manual therapy, homeopathy and Chinese medicine. This shows that there are similar recommended therapies found across different CPGs, perhaps indicating that the research regarding these CAM therapies in the context of headache/migraine is largely in agreement with one another. Additionally, we provide a legend in Fig. 2 that can support the recommendations that healthcare professionals provide to their patients suffering from headache and/or migraine disorders. This legend indicates that three of the included CPGs either have an average appraisal score or average overall assessment of 4.0 or higher for the CAM section of the CPG, and seven of the included CPGs have both an average appraisal score and average overall assessment of 4.0 or higher for the CAM section of the CPG. Healthcare providers can consult Fig. 2 to identify common CAM therapies recommended for headache and/or migraine, in addition to CAM therapies that are recommended by higher quality CPGs.

### Average appraisal scores, average overall assessments and recommendations regarding use of guidelines: overall guideline (Table 2)

The average appraisal scores for each of the 13 CPGs ranged from 3.5 to 6.3 on the seven-point Likert scale (where 7 equals strongly agree that the item is met); nine CPGs achieved or exceeded an average appraisal score of 4.0, and 3 CPGs achieved or exceeded an average appraisal score of 5.0. Average overall assessments for the 13 CPGs ranged between 3.0 (lowest) and 6.0 (highest), including 10 CPGs equalling or exceeding a score of 4.0, and 5 CPGs equalling or exceeding a score of 5.0.

**Table 2:** Average appraisal scores and average overall assessments of each guideline

Guideline	Metric	Appraiser 1	Appraiser 2	Average	Standard Deviation
Ren 2020 [28] (Overall)	Appraisal Score	4.7	4.5	4.6	0.1
	Overall Assessment	5.0	4.0	4.5	0.7
Ren 2020 [28] (CAM Section)	Appraisal Score	4.7	4.5	4.6	0.1
	Overall Assessment	5.0	4.0	4.5	0.7
Araki 2019 [29] (Overall)	Appraisal Score	4.0	3.4	3.7	0.4
	Overall Assessment	4.0	3.0	3.5	0.7
Araki 2019 [29] (CAM Section)	Appraisal Score	3.9	3.2	3.6	0.5
	Overall Assessment	4.0	3.0	3.5	0.7
Al Khaled 2017 [32] (Overall)	Appraisal Score	4.4	4.2	4.3	0.1
	Overall Assessment	4.0	4.0	4.0	0.0
Al Khaled 2017 [32] (CAM Section)	Appraisal Score	3.7	3.7	3.7	0.0
	Overall Assessment	3.0	4.0	3.5	0.7
Moisset 2016 [34] (Overall)	Appraisal Score	4.0	3.9	4.0	0.1
	Overall Assessment	5.0	3.0	4.0	1.4
Moisset 2016 [34] (CAM Section)	Appraisal Score	3.7	3.1	3.4	0.4
	Overall Assessment	5.0	3.0	4.0	1.4
Robbins 2016 [35] (Overall)	Appraisal Score	5.0	4.9	5.0	0.1
	Overall Assessment	5.0	5.0	5.0	0.0
Robbins 2016 [35] (CAM Section)	Appraisal Score	4.7	4.3	4.5	0.3
	Overall Assessment	4.0	5.0	4.5	0.7
Becker 2015 [36] (Overall)	Appraisal Score	4.9	4.6	4.8	0.2
	Overall Assessment	6.0	4.0	5.0	1.4
Becker 2015 [36] (CAM Section)	Appraisal Score	4.4	3.9	4.2	0.4
	Overall Assessment	6.0	4.0	5.0	1.4
Bendtsen 2012 [38] (Overall)	Appraisal Score	3.5	3.4	3.5	0.1
	Overall Assessment	3.0	3.0	3.0	0.0
Bendtsen 2012 [38] (CAM Section)	Appraisal Score	3.0	2.7	2.9	0.2
	Overall Assessment	3.0	3.0	3.0	0.0
Holland 2012 [39] (Overall)	Appraisal Score	4.4	4.2	4.3	0.1
	Overall Assessment	6.0	4.0	5.0	1.4
Holland 2012 [39] (CAM Section)	Appraisal Score	4.3	4.0	4.2	0.2
	Overall Assessment	6.0	4.0	5.0	1.4
NICE 2012 [40] (Overall)	Appraisal Score	6.3	6.2	6.3	0.1
	Overall Assessment	6.0	6.0	6.0	0.0
NICE 2012 [40] (CAM Section)	Appraisal Score	6.0	5.9	6.0	0.1
	Overall Assessment	6.0	6.0	6.0	0.0
Pringsheim 2012 [41] (Overall)	Appraisal Score	6.4	6.0	6.2	0.3
	Overall Assessment	6.0	6.0	6.0	0.0
Pringsheim 2012 [41] (CAM Section)	Appraisal Score	5.8	5.3	5.6	0.4
	Overall Assessment	6.0	6.0	6.0	0.0
Sarchielli 2012 [43] (Overall)	Appraisal Score	3.7	3.6	3.7	0.1
	Overall Assessment	5.0	4.0	4.5	0.7
Sarchielli 2012 [43] (CAM Section)	Appraisal Score	3.3	3.2	3.3	0.1
	Overall Assessment	4.0	4.0	4.0	0.0
Vukovic 2012 [44] (Overall)	Appraisal Score	3.7	3.3	3.5	0.3
	Overall Assessment	4.0	3.0	3.5	0.7
Vukovic 2012 [44] (CAM Section)	Appraisal Score	3.3	3	3.2	0.2
	Overall Assessment	3.0	3.0	3.0	0.0
Bryans 2011 [45] (Overall)	Appraisal Score	4.5	4.6	4.6	0.1
	Overall Assessment	5.0	4.0	4.5	0.7
Bryans 2011 [45] (CAM Section)	Appraisal Score	4.5	4.3	4.4	0.1
	Overall Assessment	5.0	4.0	4.5	0.7

### Average appraisal scores, average overall assessments and recommendations regarding use of guidelines: CAM sections (Table 2)

Average appraisal scores across the 13 CPGs ranged from 2.9 to 6.0 on the seven-point Likert scale (where 7 equals strongly agree that the item is met); seven CPGs achieved or exceeded an average appraisal score of 4.0, and 2 CPGs achieved or exceeded an average appraisal score of 5.0. For the average overall assessments, the 13 CPGs ranged between 3.0 (lowest) and 6.0 (highest), including 9 CPGs with a score of at least 4.0, and 4 CPGs with a score of at least 5.0.

### Overall recommendations: overall guideline (Table 3)

Appraisers agreed in their overall recommendation for 7 of 13 CPGs including 1 “No” [38], 2 “Yes with modifications” [29, 44], and 4 “Yes” [35, 40, 41, 45]. Of the remaining 6 CPGs, 1 was rated by the two appraisers as “No” and “Yes” respectively [34], while 5 CPGs were rated as “Yes” and “Yes with modifications” respectively [28, 32, 36, 39, 43].

**Table 3:** Overall recommendations for use of appraised guidelines

	Overall Guideline		CAM Section	
Guideline	Appraiser 1	Appraiser 2	Appraiser 1	Appraiser 2
Ren 2020 [28]	Yes	Yes with Modifications	Yes	Yes with Modifications
Araki 2019 [29]	Yes with Modifications	Yes with Modifications	Yes with Modifications	Yes with Modifications
Al Khaled 2017 [32]	Yes with Modifications	Yes	No	Yes
Moisset 2016 [34]	Yes	No	Yes	No
Robbins 2016 [35]	Yes	Yes	Yes with Modifications	Yes
Becker 2015 [36]	Yes	Yes with Modifications	Yes	Yes with Modifications
Bendtsen 2012 [38]	No	No	No	No
Holland 2012 [39]	Yes	Yes with Modifications	Yes	Yes with Modifications
NICE 2012 [40]	Yes	Yes	Yes	Yes
Pringsheim 2012 [41]	Yes	Yes	Yes	Yes
Sarchielli 2012 [43]	Yes	Yes with Modifications	Yes with Modifications	Yes with Modifications
Vukovic 2012 [44]	Yes with Modifications	Yes with Modifications	No	Yes with Modifications
Bryans 2011 [45]	Yes	Yes	Yes	Yes

### Overall recommendations: CAM sections (Table 3)

Appraisers agreed in their overall recommendation for 6 of 13 CPGs including 1 “No” [38], 2 “Yes with modifications” [29, 43], and 3 “Yes” [40, 41, 45]. Of the remaining 7 CPGs, one was rated by the two appraisers as “No” and “Yes with modifications” respectively [44], while 4 CPGs were rated as “Yes” and “Yes with modifications” respectively [28, 35, 36, 39] and 2 were rated as No and Yes [32, 34].

### Scaled domain percentage quality assessment (Table 4)

With regards to scaled domain percentages of the overall CPG, scope and purpose scores ranged from 63.9 to 100.0%, stakeholder involvement scores ranged from 22.2 to 86.1%, rigour of development scores ranged from 13.5 to 91.7%, clarity of presentation scores ranged from 66.7 to 100.0%, applicability scores ranged from 6.3 to 72.9%, and editorial independence scores ranged from 0.0 to 100.0%. With regards to scaled domain percentages of the CAM guideline sections, scope and purpose scores ranged from 61.1 to 100.0%, stakeholder involvement scores ranged from 13.9 to 17.8%, rigour of development scores ranged from 9.4 to 85.4%, clarity of presentation scores ranged from 50.0 to 100.0%, applicability scores ranged from 0.0 to 68.8%, and editorial independence scores ranged from 0.0 to 100.0%.

**Table 4:** Scaled domain percentages for appraisers of each guideline

Guideline		Domain score (%)					
		Scope and Purpose	Stakeholder Involvement	Rigour of Development	Clarity of Presentation	Applicability	Editorial Independence
Ren 2020 [28]	Overall Guideline	94.4	77.8	54.2	69.4	37.5	37.5
	CAM Section	94.4	77.8	54.2	69.4	37.5	37.5
Araki 2019 [29]	Overall Guideline	66.7	36.1	41.7	88.9	18.8	29.2
	CAM Section	75.0	16.7	44.8	66.7	18.8	29.2
Al Khaled 2017 [32]	Overall Guideline	91.7	61.1	44.8	94.4	14.6	58.3
	CAM Section	91.7	36.1	38.5	72.2	6.3	58.3
Moisset 2016 [34]	Overall Guideline	97.2	61.1	32.3	80.6	12.5	45.8
	CAM Section	91.7	44.4	27.1	61.1	4.2	45.8
Robbins 2016 [35]	Overall Guideline	80.6	36.1	79.2	91.7	14.6	100.0
	CAM Section	80.6	27.8	68.8	75.0	10.4	100.0
Becker 2015 [36]	Overall Guideline	83.3	77.8	47.9	83.3	39.6	87.5
	CAM Section	80.6	52.8	40.6	63.9	31.3	87.5
Bendtsen 2012 [38]	Overall Guideline	94.4	44.4	13.5	75.0	27.1	41.7
	CAM Section	80.6	33.3	9.4	50.0	16.7	41.7
Holland 2012 [39]	Overall Guideline	83.3	22.2	65.6	66.7	8.3	95.8
	CAM Section	83.3	13.9	60.4	66.7	6.3	95.8
NICE 2012 [40]	Overall Guideline	100.0	83.3	91.7	97.2	72.9	75.0
	CAM Section	100.0	66.7	85.4	97.2	68.8	75.0
Pringsheim 2012 [41]	Overall Guideline	94.4	86.1	86.5	100.0	70.8	87.5
	CAM Section	94.4	47.2	79.2	100.0	54.2	87.5
Sarchielli 2012 [43]	Overall Guideline	63.9	30.6	49.0	91.7	6.3	16.7
	CAM Section	61.1	19.4	42.7	77.8	2.1	16.7
Vukovic 2012 [44]	Overall Guideline	91.7	44.4	25.0	86.1	25.0	0.0
	CAM Section	88.9	30.6	22.9	80.6	10.4	0.0
Bryans 2011 [45]	Overall Guideline	86.1	50.0	63.5	88.9	6.3	75.0
	CAM Section	86.1	41.7	62.5	91.7	0.0	75.0

### Scope and purpose

For the overall CPG, the overall objectives and health questions were generally well-defined in all CPGs. All CPGs described the goal, the disease or condition that was to be managed by the CAM therapies and the types of CAM therapies that were to be studied. The description of the population for whom the CPG was intended to apply was at times vague, as was the description of the health questions [43]. For the CAM section, the overall objective was generally well-defined in all CPGs. Across the CAM sections of CPGs, descriptions of the population to whom the CPG was meant to apply was less clear [29, 35, 43].

### Stakeholder involvement

For the overall CPG, the majority of the CPGs provided detailed characteristics for the members of the CPG development group, including information about the degrees held by each panel member, their institutional affiliations, and geographical location. Most of the CPGs did not elaborate on the views and preferences of the target population, with only a few declaring this [28, 36, 40, 41]. The majority of CPGs thoroughly defined their target users, by providing information about the type of clinician and the specialties. Only three CPGs were vague in their description of target users [29, 39, 43]. For the CAM section, CPGs scored lower across all three sections overall when compared to the overall assessment as there was less involvement of CAM specific specialists. The target users were generally the only detailed subject across all CAM sections. Only two CPGs provided detailed and thorough information to support the three criteria of this domain for both overall and CAM assessments [28, 45].

### Rigor of development

For the overall CPG, most of the CPGs used systematic methods to search for evidence according to detailed selection criteria, with the exception of four CPGs [32, 34, 38, 44]. The strengths and limitations of the body of evidence were clearly described in all CPGs except for one [38]. Some CPGs provided detail about the methods for formulating recommendations [28, 34–36, 41, 45], while other CPGs provided minimal detail [29, 32, 38, 39, 43, 44]. All CPGs formulated recommendations with considerations of some health benefits, side effects, and/or risks in formulating their recommendations, with the exception of one [28]. With the exception of one CPG [28], all CPGs provided an explicit link between their recommendations and the supporting evidence. Most of the CPGs did not provide detail or explicitly state that they were externally reviewed by experts prior to publication [29, 32, 34, 36, 38, 43–45], with five CPGs providing explicit statements [28, 35, 39–41]. Most CPGs did not include a procedure for updating the CPG [29, 34, 36, 38, 39, 43–45] and those that did only outlined their methodology vaguely [28, 32, 35, 40, 41]. For the CAM section, the majority of the scores remained the same as the overall assessment, however, many CPGs described health benefits, side effects and/or risks that influenced recommendations [32, 35, 36, 38, 43, 44], as well as the description of external review [32, 34–36, 39–41], more vaguely.

### Clarity of presentation

For the overall CPG, the majority of them offered specific and unambiguous recommendations, with the exception of four that lacked details, such as identification of intent/purpose and relevant population [28, 34, 38, 39]. All CPGs presented different options for the management of the condition and were easily identifiable. The generally overall high scores contributed to the high scaled domain percentage [28, 29, 32, 34–36, 38–41, 43, 45]. For the CAM section, the scores were lower for the specificity and unambiguity of the CAM recommendations for several CPGs [29, 32, 35, 36, 38, 43]. Most of the CAM sections provided different management options and were easily identifiable, with the expectation of one [34].

### Applicability

For the overall CPG, few CPGs vaguely discussed facilitators and barriers to implementation of the recommendations [28, 35, 40, 41]. Four CPGs included advice and/or tools, such as flowcharts and algorithms, to support implementation of the recommendations [28, 36, 40, 41]. Four CPGs vaguely addressed the resource implications of implementing the recommendations [28, 29, 41, 44], with one addressing it in more detail [40]. Six CPGs [32, 34, 36, 38, 40, 41] provided vague monitoring and auditing criteria, while 7 CPGs contained little to no such information [28, 29, 35, 39, 43–45]. For the CAM section, the scores remained similar to the overall assessment, or the scores were lower across all the applicability criteria, with some related to monitoring and auditing criteria being notably lower than the overall score [28, 44].

### Editorial Independence

In the overall CPG, the reporting of the funding source or competing interests of the members of the CPG development panel varied. Several CPGs did not report funding sources or how these sources influenced the CPG development [29, 34, 38, 43, 44]. All the CPGs addressed competing interests, with some varying in the detail regarding how potential competing interests were identified or considered, or how they may have influenced the CPG development process [28, 29, 32, 38, 43, 45]. Some CPGs described in a clear statement that there were no competing interests in the development of the CPGs [28, 29, 45]. One CPG did not address competing interests [44]. For the CAM section, the scores were identical to that of the overall assessment, as the nature of the items in the editorial independence domain are that they pertain to the overall CPG.

## Discussion

The purpose of this study was to identify the quantity and assess the quality of CAM recommendations in CPGs for the treatment and/or management of headache and migraine. This research seeks to identify credible, knowledge-based resources which healthcare professionals can use in their discussions and decisions with patients about the use of CAM. We identified 21 eligible CPGs published between 2009 and 2020, of which 13 CPGs made CAM therapy recommendations. The quality of CPGs containing CAM recommendations was assessed by the 23-item AGREE II instrument, which varied widely across CPGs overall and by domain. The overall CPG assessment included three CPGs scoring 5.0 or higher in both average appraisal score and average overall assessment [35, 40, 41]. In assessing the CAM section of each CPG, two CPGs scored 5.0 or higher in both average appraisal score and average overall assessment [40, 41].

To our knowledge, this is the first study to assess the quantity and quality of CAM therapies recommendations in headache and/or migraine CPGs. In this study, the scaled domain percentages for the overall CPGs from highest to lowest were clarity of presentation (66.7%), scope and purpose (63.9%), stakeholder involvement (22.2%), rigour of development (13.5%), applicability (6.3%) and editorial independence (0.0%). The scaled domain percentages for the CAM section of the CPGs from highest to lowest were scope and purpose (61.1%), clarity of presentation (50.0%), stakeholder involvement (13.9%), rigor of development (9.4%), and editorial independence (0.0%) and applicability (0.0%).

Published studies on similar topics relating to CPG quality assessment exist, allowing us to draw comparisons. A study appraising the methodological quality of CPGs for headache, which included some CPGs providing traditional Chinese medicine recommendations, found that, among 23 CPGs published between 1998 and 2014, the scaled domain percentages were similarly ordered from highest (scope and purpose 52.1%) to lowest (editorial independence 24.2%) [49]. One study assessing the quality of CPGs recommending herbal medicines, acupuncture, and spinal manipulation, which are recommended by several CPGs included in the present review, reported quality scores similar to our study, with clarity of presentation being the highest scaled domain percentage (85.3%) and applicability the lowest (20.7%) [50]. Another study examining the quality of CAM recommendations in 15 arthritis CPGs found that the highest scoring domain was clarity of presentation (94.1%), and the lowest was applicability (33.3%) [51]. One study assessed the quality of CAM recommendations across cancer-related pain CPGs, and found that the highest scored domain for both the overall CPG and the CAM section was scope and purpose at 88.1 and 88.1% respectively, while the lowest scored domain was applicability at 21.0 and 8.5% respectively [52]. Finally, two other studies assessing the quality of CAM recommendations across multiple sclerosis and low back pain CPGs respectively found that the highest scoring domains were clarity of presentation and scope of purpose, while the lowest scoring domains were editorial independence and stakeholder involvement [53, 54]. Therefore, the variable quality of headache and migraine CPGs in the context of CAM recommendations is not unique, as the same phenomenon has been observed across CPGs for a variety of diseases and conditions.

This study described the quantity and quality of headache and/or migraine CPGs that included CAM recommendations, revealing that several CPGs can be used to support informed decision-making among healthcare professionals to better inform and support their patients. However, the quality of these recommendations can be improved. Randomized controlled trials used to inform the development of CPGs suffer from several limitations and discrepancies, including insufficient sample sizes, lack of funding and biased grant review processes, making it difficult to formulate conclusions regarding their efficacy [55, 56]. This can be seen in our study, where three CPGs that scored higher in overall assessment also scored higher in their CAM sections than that of CPGs with lower overall assessment scores [28, 40, 41]. Also, there is an increasing interest in and prevalence of CAM use among patients suffering from headache or migraine, but there are discrepancies between patient-reported use and the available evidence-based for CAM therapies [14–16, 56]. Future directions worth exploring given the present review’s findings include the further investigation of how patient preference and experience relating to CAM therapies can better be incorporated into headache/migraine CPGs. Approximately 82% of individuals experiencing headache disorders use CAM therapies [57]. Although healthcare professionals prefer recommending evidence-based therapies, many may be hesitant to recommend even evidence-based CAM therapies due to their lack of knowledge/training, concerns regarding dosing, or personal biases against CAM [21, 58, 59]. Therefore, our finding of lower quality recommendations across most CPGs in combination with the fact that there is increasing interest in CAM therapies among patients further justifies the need to incorporate recommendations for evidence-based and commonly used CAMs in future headache and migraine CPGs.

### Strengths and limitations

One strength of this study is the use of a comprehensive systematic review to identify eligible CPGs for the treatment and/or management of headache and/or migraine. Another strength is the use of the validated AGREE II instrument, which is the internationally-accepted gold standard for appraising the quality of CPGs [27]. CPGs were independently assessed by two appraisers instead of four as recommended by the AGREE II instrument to optimize reliability, thus this may limit the interpretation of the findings. In an effort to mitigate this and standardize scoring, JYN, CH and the other research assistant conducted an initial pilot-test during which they each appraised three independent CPGs, then discussed the results to achieve consensus on how to apply the AGREE II instrument. Following appraisal of the 13 CPGs, JYN met with CH and the other research assistant to resolve any uncertainties through discussion without inordinately modifying legitimate discrepancies. Another limitation includes the fact that our inclusion criteria was limited to CPGs that were only published in the English language; this increases the possibility that there could be omissions of other traditional medicine therapy recommendations that originate from different (i.e. non-English speaking) regions of the world, which could also be greatly influenced by the culture of that region. Furthermore, the development of CPGs from other regions of the world could be influenced by the availability of stakeholders and medical resources, indicating that regional and cultural differences can affect the recommendation grade. For example, there could be articles published in Asian countries, which would have more information about traditional Asian medicine, given the higher frequency of use.

## Conclusions

This study identified 13 CPGs published between 2009 and 2020 which included CAM therapy recommendations for the treatment and/or management of headache and/or migraine. The CPGs included in this study provided CAM-specific recommendations related to subsets of CAM therapies, including dietary supplements, oxygen therapy, herbal medicine, electrotherapy, and acupuncture. The AGREE II instrument was used for the appraisal of these CPGs, identifying the varied quality within and across them. Some CPGs achieved higher AGREE II scores and favourable overall recommendations, thus patients and healthcare professionals could use them as a basis for discussion about the use of these CAM therapies to treat or manage headache or migraine. CPGs that achieved lower scaled domain percentage and overall recommendations could be improved in future updates according to the criteria as outlined in the AGREE II instrument.

## Supplementary Information

**Additional file 1.** MEDLINE Search Strategy for Headache and Migraine Clinical Practice Guidelines Executed April 17, 2020

**Additional file 2.** Modified AGREE II Questions Used to Guide Scoring of CAM Sections of Each Guideline

**Additional file 3.** List of Excluded Full-Text Items

## Data Availability

All relevant data are included in this manuscript.

## Abbreviations

AGREE II: Appraisal of Guidelines for Research & Evaluation II
CAM: Complementary and Alternative Medicine
CPG: Clinical Practice Guideline
ICHD: International Classification of Headache Disorders
NCCIH: National Center for Complementary and Integrative Health
PICO: Patients, Intervention, Comparison and Outcomes
PRISMA: Preferred Reporting Items for Systematic Reviews and Meta-Analyses
NSAIDS: Nonsteroidal Anti-Inflammatory Drugs
