# Supplementary material for: Headache and migraine clinical practice guidelines: a systematic review and assessment of complementary and alternative medicine recommendations
Source: BMC Complement Med Ther. 2021 Sep 22;21:236. doi: 10.1186/s12906-021-03401-3 (PMC8456672; doi:10.1186/s12906-021-03401-3)
Supplement: Supplementary file 1 — Additional file 1. MEDLINE Search Strategy for Headache and Migraine Clinical Practice Guidelines Executed April 17, 2020 [file 12906_2021_3401_MOESM1_ESM.docx]

## Supplementary File 1: MEDLINE Search Strategy for Headache and Migraine Clinical Practice Guidelines Executed April 17, 2020

| Database: Ovid MEDLINE(R) and Epub Ahead of Print, In-Process & Other Non-Indexed Citations, Daily and Versions(R) <1946 to April 16, 2020>  Search Strategy:  --------------------------------------------------------------------------------  1 Post-Traumatic Headache/ or Headache Disorders, Primary/ or Tension-Type Headache/ or Headache Disorders/ or headache.mp. or Headache/ or Headache Disorders, Secondary/ or Cluster Headache/ or Post-Dural Puncture Headache/ (82919)  2 migraine.mp. or Migraine Disorders/ (38351)  3 or/1-2 (104231)  4 limit 3 to ("all infant (birth to 23 months)" or "all child (0 to 18 years)" or "newborn infant (birth to 1 month)" or "infant (1 to 23 months)" or "preschool child (2 to 5 years)" or "child (6 to 12 years)" or "adolescent (13 to 18 years)") (23106)  5 3 not 4 (81125)  6 limit 5 to (english language and humans and yr="2009 -2020" and (guideline or practice guideline)) (47)  *************************** |
| --- |

## 
