# Supplementary material for: Headache and migraine clinical practice guidelines: a systematic review and assessment of complementary and alternative medicine recommendations
Source: BMC Complement Med Ther. 2021 Sep 22;21:236. doi: 10.1186/s12906-021-03401-3 (PMC8456672; doi:10.1186/s12906-021-03401-3)
Supplement: Supplementary file 3 — Additional file 3. List of Excluded Full-Text Items [file 12906_2021_3401_MOESM3_ESM.docx]

**Supplementary File 3: List of Excluded Full-Text Items**

Reason: Irretrievable

1. Hao L, Hui L,Yangyang W, Sha Y, Wenjie X. Traditional Chinese medicine treatment guideline for primary headache disorders. 2018. <https://www.guidelinecentral.com/summaries/traditional-chinese-medicine-treatment-guideline-for-primary-headache-disorders/#section-society>

Reason: Guideline Summary

1. Haag G, Diener HC, May A, Meyer C, Morck H, Straube A, Wessely P, Evers S. Self-medication of migraine and tension-type headache: Summary of the evidence-based recommendations of the Deutsche Migräne und Kopfschmerzgesellschaft (DMKG), the Deutsche Gesellschaft für Neurologie (DGN), the Österreichische Kopfschmerzgesellschaft (ÖKSG) and the Schweizerische Kopfwehgesellschaft (SKG). The journal of headache and pain. 2011 Apr;12(2):201-17. <https://doi.org/10.1007/s10194-010-0266-4>.
